# USP14 Regulates Cancer Cell Growth in a Fatty Acid Synthase-Independent Manner

**DOI:** 10.3390/ijms222413437

**Published:** 2021-12-14

**Authors:** Ji Su Yang, Naeun Yoon, Mingyu Kong, Byung Hwa Jung, Hyunbeom Lee, Jinyoung Park

**Affiliations:** 1 Molecular Recognition Research Center, Korea Institute of Science and Technology, Seoul 02792, Korea; jane6263@kist.re.kr (J.S.Y.); betteryun@kist.re.kr (N.Y.); 119524@kist.re.kr (M.K.); jbhluck@kist.re.kr (B.H.J.); 2 Department of Life Sciences, College of Life Sciences and Biotechnology, Korea University, Seoul 02841, Korea; 3 College of Pharmacy, Sookmyung Women’s University, Seoul 04310, Korea; 4 Department of Biomedical and Phamaceutical Sciences, Kyung Hee University, Seoul 02453, Korea; 5 Division of Bio-Medical Science & Technology, KIST-School, Korea University of Science and Technology (UST), Seoul 02792, Korea; 6 Department of HY-KIST Bio-convergence, Hanyang University, Seoul 04763, Korea

**Keywords:** fatty acid synthase, USP14, cancer

## Abstract

Fatty acid synthase (FASN) plays an important role in cancer development, providing excess lipid sources for cancer growth by participating in de novo lipogenesis. Although several inhibitors of FASN have been developed, there are many limitations to using FASN inhibitors alone as cancer therapeutics. We therefore attempted to effectively inhibit cancer cell growth by using a FASN inhibitor in combination with an inhibitor of a deubiquitinating enzyme USP14, which is known to maintain FASN protein levels in hepatocytes. However, when FASN and USP14 were inhibited together, there were no synergistic effects on cancer cell death compared to inhibition of FASN alone. Surprisingly, USP14 rather reduced the protein levels and activity of FASN in cancer cells, although it slightly inhibited the ubiquitination of FASN. Indeed, treatment of an USP14 inhibitor IU1 did not significantly affect FASN levels in cancer cells. Furthermore, from an analysis of metabolites involved in lipid metabolism, metabolite changes in IU1-treated cells were significantly different from those in cells treated with a FASN inhibitor, Fasnall. These results suggest that FASN may not be a direct substrate of USP14 in the cancer cells. Consequently, we demonstrate that USP14 regulates proliferation of the cancer cells in a fatty acid synthase-independent manner, and targeting USP14 in combination with FASN may not be a viable method for effective cancer treatment.

## 1. Introduction

Fatty acid synthase (FASN) is an essential enzyme in de novo lipogenesis that catalyzes the synthesis of palmitate, a 16-carbon chain saturated fatty acid (FA), from acetyl-CoA and malonyl-CoA in the presence of nicotinamide adenine dinucleotide phosphate hydrogen (NADPH) [1,2,3,4]. In normal cells, FASN expression is commonly low because the cells are supplied with FAs from nutrients and lipids stored in adipose tissue or liver. On the other hand, tumor cells require more lipid sources for cancer growth and survival, which is highly dependent on de novo lipogenesis through FASN [5,6]. Therefore, FASN is overexpressed in various cancer types such as breast, ovarian, liver, and prostate cancer and serves as a potential oncogene, indicating that FASN may be an attractive therapeutic target for cancer [7,8,9,10].

Many studies have shown that inhibition of FASN activity by pharmacological drugs and siRNAs induces apoptosis of cancer cells in vitro [11]. As a result, various FASN inhibitors were developed as anticancer drugs, but most of them failed in clinical trials with the exception of TVB-2640, which is currently undergoing phase 2 clinical trials in combination with other drugs for HER2+ breast cancer [12,13]. Most FASN inhibitors have unexpected toxicities in vivo that may be attributed to a lack of target selectivity, pharmacological side effects, and metabolic flexibility of cancer cells [14,15,16,17,18,19]. Indeed, the de novo lipogenesis pathway is complexly regulated by numerous enzymes at multiple steps. Therefore, when the FASN activity is inhibited solely using a FASN inhibitor, compensatory adaptive responses including an increase in the expression of FASN itself are likely to occur, resulting in mitigated efficacy of the FASN inhibitor in vivo [4,11].

FASN protein levels can be regulated by the ubiquitination/deubiquitination pathway. It is well known that USP2a maintains the stability of FASN through deubiquitination in androgen-dependent prostate cancer. USP2a protects FASN from proteasomal degradation, inhibiting apoptosis and increasing cancer cell proliferation [20]. In contrast, the COP1 E3 ligase binds to FASN via SH2 adaptor protein and promotes FASN ubiquitination. COP1 phosphorylated by p38 MAP kinase accumulates in the cytoplasm to form the SH2-COP1-FASN complex, leading to degradation of FASN [21]. Recently, Liu et al. reported that Usp14 increases FASN stability in mouse primary hepatocytes (MPHs). They demonstrated that overexpression of Usp14 controls FASN levels, promoting blood glucose and insulin levels and liver and plasma TG levels, and increases liver weight in normal mice, whereas knockdown of Usp14 ameliorates hepatosteatosis in db/db mice [22].

USP14 is one of the proteasomal-associated deubiquitinating enzymes (DUBs), which prevents substrate degradation by cleaving the ubiquitin chain of the substrate as soon as it is attached to the proteasome [23]. USP14 expression is upregulated in various cancers, and the oncogenic effect of USP14 has been widely reported [24]. Based on these reports, many studies have suggested that USP14 inhibitors alone or in combination with other anticancer drugs might be potent cancer therapeutics [25,26,27,28,29].

In this study, we hypothesized that the compensatory adaptive response of lipid metabolism by FASN inhibitors in cancer cells can be counteracted by reducing FASN levels using IU1, a USP14 inhibitor, and therefore inhibiting FASN and USP14 together will dramatically suppress the cancer cell growth. However, inhibition of both FASN and USP14 had no significant synergistic effect on cancer cell proliferation and, surprisingly, it was confirmed that USP14 negatively regulates the protein level and activity of FASN in cancer cells. In fact, IU1 treatment did not significantly affect FASN levels. Through untargeted metabolomic profiling, we found that a significant number of metabolites altered by USP14 inhibition and FASN inhibition were different, respectively. This can be interpreted as meaning that inhibition of USP14 does not help in terms of modulating the metabolic pathways in the cancer cells by FASN. Therefore, we conclude that USP14 inhibition does not compensate for the weakness of the FASN inhibitor and suggest that the use of both inhibitors together as cancer therapeutics is undesirable.

## 2. Results

### 2.1. Inhibition of USP14 Has No Synergistic Effects on Cancer Cell Proliferation Reduced by FASN Inhibition

**Figure 1:**
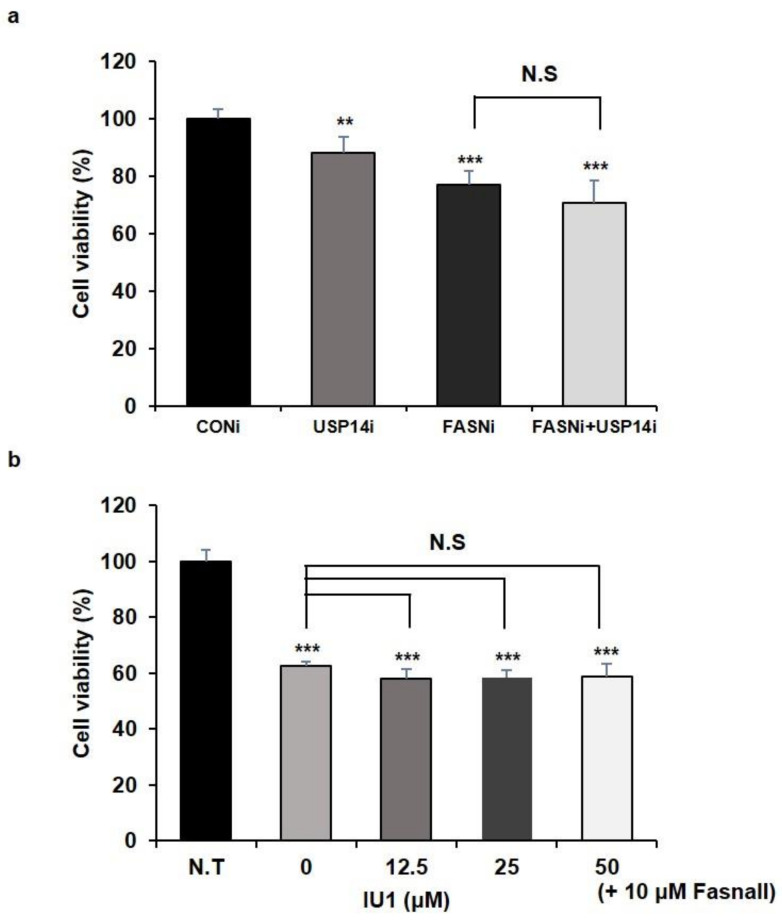
Inhibition of USP14 and FASN together has no synergistic effect on cancer cell proliferation. (**a**,**b**) Cell viability was measured by WST-1 assay. LNCaP cells were (**a**) transfected with siRNA targeting *FASN* (FASNi) or *USP14* (USP14i) for 72 h or (**b**) treated with Fasnall or IU1 at indicated concentration for 48 h. Data are shown as mean SD and determined by three independent repeated experiments (**; *p* < 0.01, ***; *p* < 0.001 *t*-test). N.S; nonspecific, N.T; nontreated.

It is well known that FASN is a metabolic oncogene, and overexpression of FASN is commonly observed in cancer cells. In addition to USP2a, which is well known as a DUB that regulates the level of FASN, a recent study showed that Usp14 significantly contributes to the development of hepatosteatosis by maintaining the stability of FASN in MPHs [7,20]. However, the effect of USP14 on the function or stability of FASN in cancer cells is completely unknown. Since Usp14 regulates the level of FASN in MPHs, we predicted that knockdown or inhibition of FASN and USP14 together would have a synergistic effect in reducing the proliferation of cancer cells. We performed a cell proliferation assay under two conditions: First, we transfected human prostate cancer cell lines LNCaP cells with siRNAs targeting *FASN* or *USP14* (Figure S1a,b), either individually or together. As reported previously, cell proliferation was reduced by FASN or USP14 knockdown, respectively [7,30]. However, there was no synergistic effect on reducing cell proliferation by FASN and USP14 knockdown together (Figure 1a). We also treated LNCaP cells with USP14 inhibitor IU1 or FASN inhibitor Fasnall individually or together. Cell viability was measured at 24 and 48 h after treatment with IU1 or FASN. When IU1 was treated at concentrations of 12.5, 25, and 50 μM, significant inhibition of cancer cell viability was observed only after treatment with 50 μM IU1 for 48 h (Figure S2a). In the case of Fasnall, cell viability decreased after treatment for 24 h in a concentration-dependent manner (Figure S2b). Based on these results, we examined whether there was a synergistic effect on the reduction of cell viability when several concentrations of IU1 were simultaneously treated after treatment with 10 μM Fasnall for 48 h. However, we did not find any synergistic effect on cancer cell viability (Figure 1b). These results indicated that individual inhibition of USP14 and FASN mildly reduced cancer cell proliferation, but contrary to our expectation, no synergistic effect was confirmed when both were inhibited.

### 2.2. USP14 Negatively Regulates the Levels and Activity of FASN in Cancer Cells

**Figure 2:**
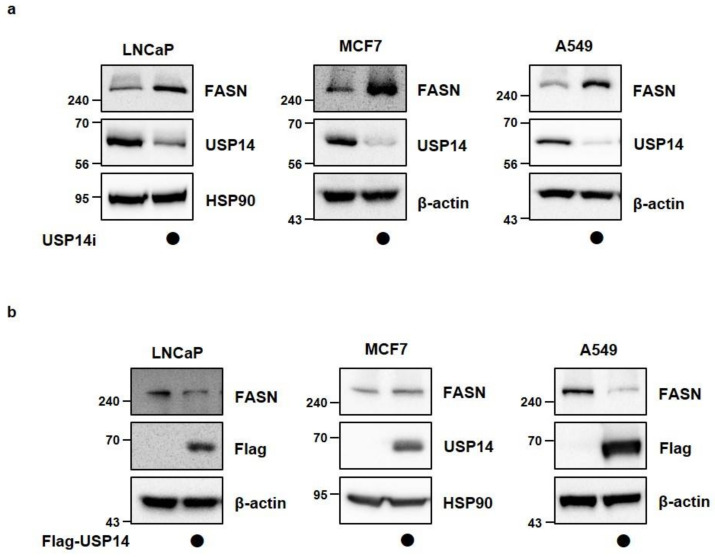
USP14 negatively regulates FASN levels in cancer cell lines. (**a**) LNCaP, MCF7, and A549 cells were transfected with CONi or USP14i for 48 h. Cell lysates were immunoblotted with the indicated antibodies. HSP90 and β-actin were used as a loading control. (**b**) LNCaP, MCF7, and A549 cells were transfected with Flag-USP14 for 24 h. Cell lysates were immunoblotted with the indicated antibodies.

**Figure 3:**
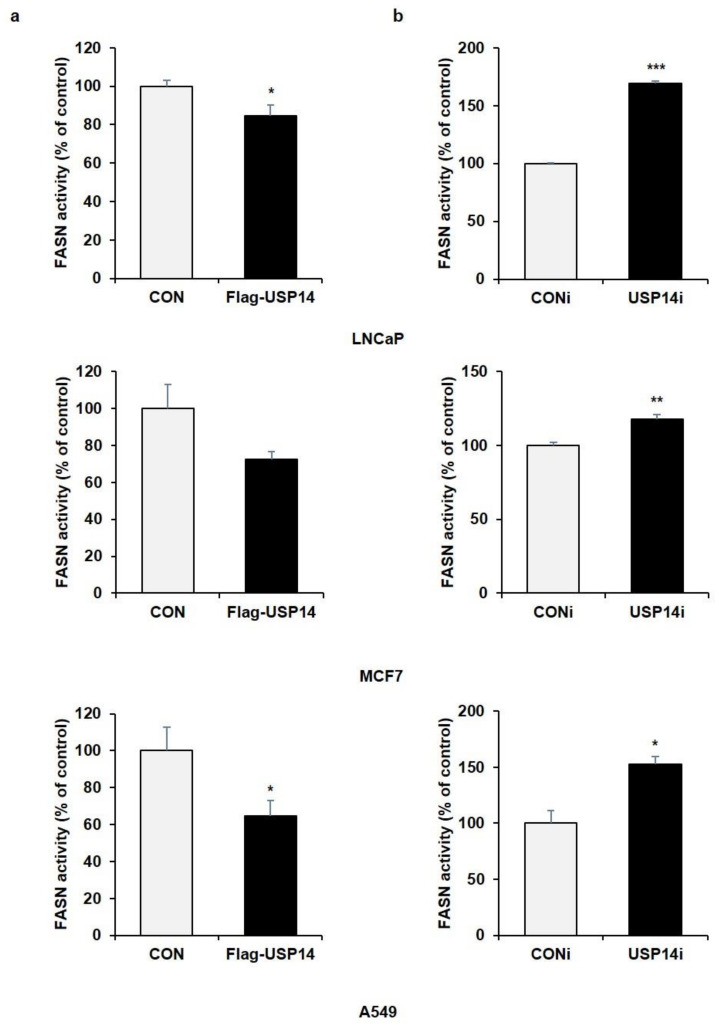
USP14 negatively regulates FASN activity in cancer cell lines. FASN activity was measured in LNCaP, MCF7, and A549 cells transfected with (**a**) Flag-USP14 for 24 h or (**b**) USP14i for 48 h. (*; *p* < 0.05, **; *p* < 0.01, ***; *p* < 0.001 *t*-test).

To determine whether USP14 plays a role as a regulator of endogenous FASN levels in cancer cells in the same manner as MPHs, we transfected various cancer cell lines with siRNA targeting USP14. In LNCaP, MCF7 (human breast cancer cell), and A549 cell (human lung cancer cell), endogenous FASN protein levels were increased by USP14 knockdown contrary to the results in MPHs (Figure 2a). In addition, USP14 overexpression reduced the FASN protein level in cancer cells (Figure 2b). Indeed, overexpression of USP2a, which is another DUB for FASN, upregulated FASN protein levels, and reduced FASN ubiquitination in LNCaP cells (Figure S3a,b). We further confirmed whether FASN enzyme activity is also regulated by USP14. As a result, USP14 overexpression reduced enzymatic activity of FASN, whereas USP14 deficiency significantly increased FASN activity in cancer cells (Figure 3a,b). These results were consistent with alteration of FASN levels by USP14 in cancer cells. Therefore, these findings demonstrated that USP14 negatively regulates protein levels and activity of FASN in cancer cells.

### 2.3. Inhibition of USP14 Does Not Significantly Affect FASN Protein Levels with IU1 in Cancer Cells

**Figure 4:**
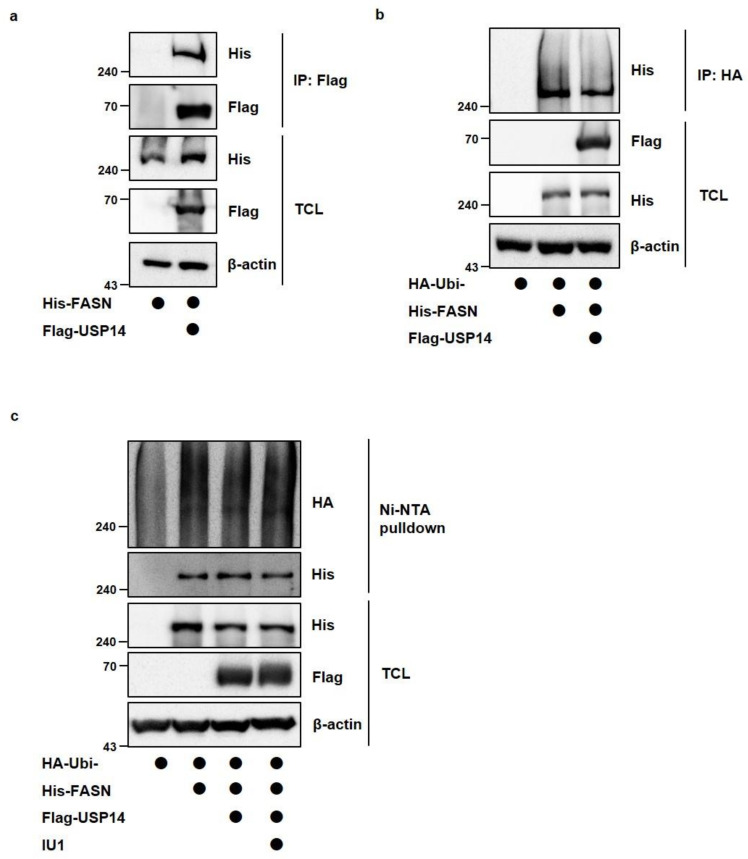
USP14 slightly reduces the ubiquitination of FASN. (**a**) HEK293T cells were transfected with His-FASN alone or in combination with Flag-USP14. Interaction between His-FASN and Flag-USP14 was detected by immunoblotting after immunoprecipitation with anti-flag antibody. (**b**) LNCaP cells were transfected with HA-ubiquitin alone or in combination with Flag-USP14 or His-FASN. MG132 treated for 6 h in cells before the harvest. FASN ubiquitination was observed by immunoblotting after immunoprecipitation with anti-HA antibody. (**c**) LNCaP cells were transfected with HA-ubiquitin alone or in combination with Flag-USP14 or His-FASN. IU1 (10 μM) treated for 24 h and MG132 treated for 6 h in cells before the harvest. FASN ubiquitination was observed using a Ni-NTA-mediated pull-down assay. TCL; total cell lysates.

**Figure 5:**
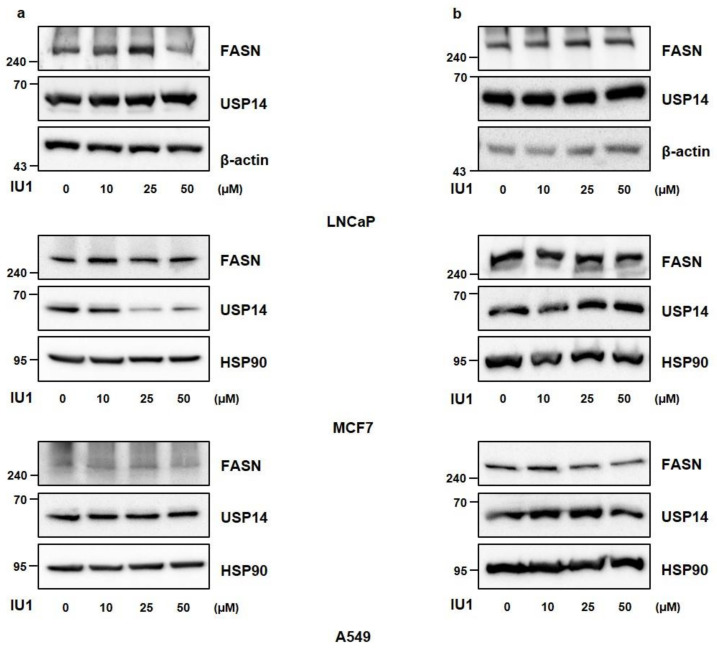
IU1 does not significantly affect FASN protein levels. (**a**,**b**) LNCaP, MCF7, and A549 cells were treated with IU1 at the indicated concentration for (**a**) 6 h or (**b**) 24 h. Western blot analysis was performed to detect FANS protein levels.

Since USP14 reduced FASN protein levels in cancer cells, it was necessary to confirm the deubiquitination of FASN by USP14. Before that, we checked the interaction between USP14 and FASN when Flag-USP14 and His-FASN were cotransfected (Figure 4a). To identify the ubiquitination of FASN, we transfected LNCaP cells with His-FASN, HA-ubiquitin, and Flag-USP14 together. As shown in Figure 4b,c, USP14 slightly reduced FASN ubiquitination. However, treatment of USP14 inhibitor IU1 did not significantly alter the ubiquitination of FASN (Figure 4c). In addition, when LNCaP, MCF7, and A549 cells were treated with different concentrations and incubation times of IU1, there was little difference in FASN levels compared to the control cells (Figure 5a,b). Taken together, these results indicated that FASN binds to USP14 but may not be a direct substrate of USP14, and the expression level of USP14 is more important in modulating FASN levels than the activity of USP14 in cancer cells.

### 2.4. Metabolomic Profiling of IU1- and Fasnall-Treated LNCaP Cells

**Figure 6:**
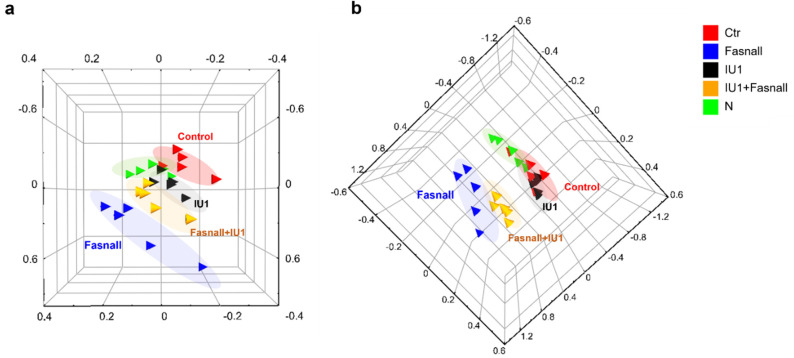
PCA plots of USP14 and FASN inhibitor-treated LNCaP cells. The score plots of principal component analysis (PCA) obtained from the metabolomic analysis of LNCaP-LN3 cells treated with FASN and USP14 inhibitors in (**a**) positive and (**b**) negative ionization modes. Green, IU1-treated group; Red, Fasnall-treated group; Blue, IU1- and Fasnall-treated group; Black, control group (with DMSO); Yellow, vehicle group (without DMSO).

Next, we wondered whether inhibition of USP14 activity affects FASN-involved lipid metabolism. To determine the simultaneous effects of FASN and USP14 inhibition on the metabolite profile, we carried out an untargeted metabolomic analysis using UPLC-Orbitrap-MS/MS in positive and negative ion modes following 24 h of exposure of LNCaP cells to each and both of Fasnall and IU1. A total of 460 and 857 features were detected in the positive and negative ion modes, respectively. A principal component analysis (PCA) was initially performed using the LC-MS/MS datasets for each peak extracted from the Compound Discoverer 2.0 to obtain the natural clustering trend for each group. As shown in Figure 6a,b, apparent clustering and separation were observed among the groups and clearly showed the difference between the Fasnall vs. IU1 treated group in both ion modes. These results clearly showed the difference in the metabolite profiles among Fasnall-treated vs. IU1-treated vs. both treated groups.

### 2.5. Altered Metabolite Identification in IU1- and Fasnall-Treated LNCaP Cells

**Figure 7:**
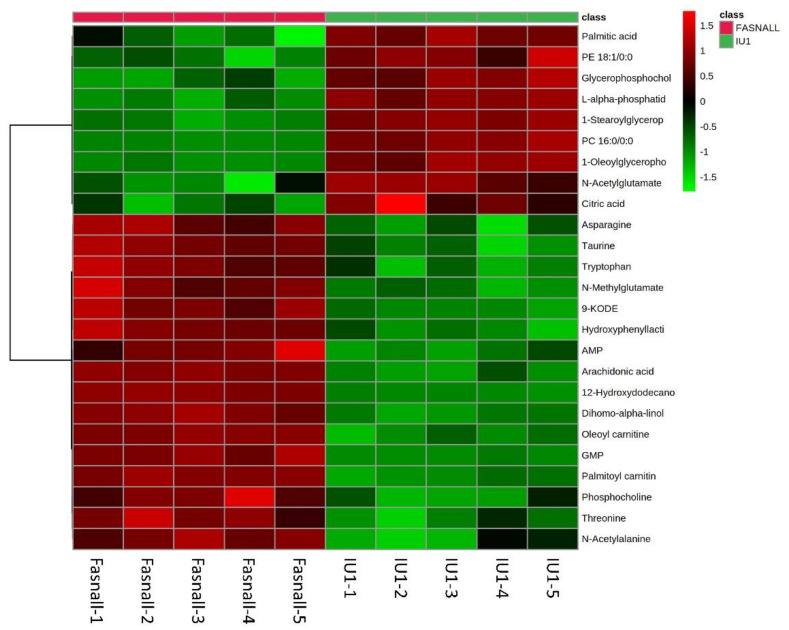
Heatmap analysis of USP14 and FASN inhibitor-treated LNCaP cells. The heatmap represents the intensities of the 25 most significantly (*p* < 0.05) altered metabolites between groups. Group is indicated at the top of the figure by red (Fasnall, *n* = 5) or green (IU1, *n* = 5). Data were sum normalized, log transformed, and autoscaled.

Among the total features obtained, we focused on identifying the significantly altered metabolites in at least one group using the METLIN, HMDB, Lipidblast, and KEGG online databases, as well as an in-house database. As a result, 57 polar and nonpolar metabolites were found and are listed in Table A1. The categories of metabolites identified include amino acids, fatty acyls, glycerophospholipids, and nucleoside analogs. We performed a hierarchical clustering heatmap analysis to visualize the metabolic significance between the Fasnall-treated and IU1-treated group. The top 25 metabolites that varied between the two groups are shown in Figure 7. The most prominent differences between them were palmitic acid, glycerophosphocholine, and citric acid. These molecules were relatively decreased in the Fasnall-treated group. However, a number of amino acids, including asparagine, tryptophan, and threonine, were relatively decreased in the IU1-treated group. These results indicate that the drugs inhibiting FASN and USP14 differently affect the metabolic pathway. Especially for palmitic acid, the levels declined with Fasnall as expected, but the levels rose with IU1. This result further confirms that USP14 negatively regulates the protein level and the activity of FASN in cancer cells.

### 2.6. Metabolic Pathway Analysis of IU1- and Fasnall-Treated LNCaP Cells

**Figure 8:**
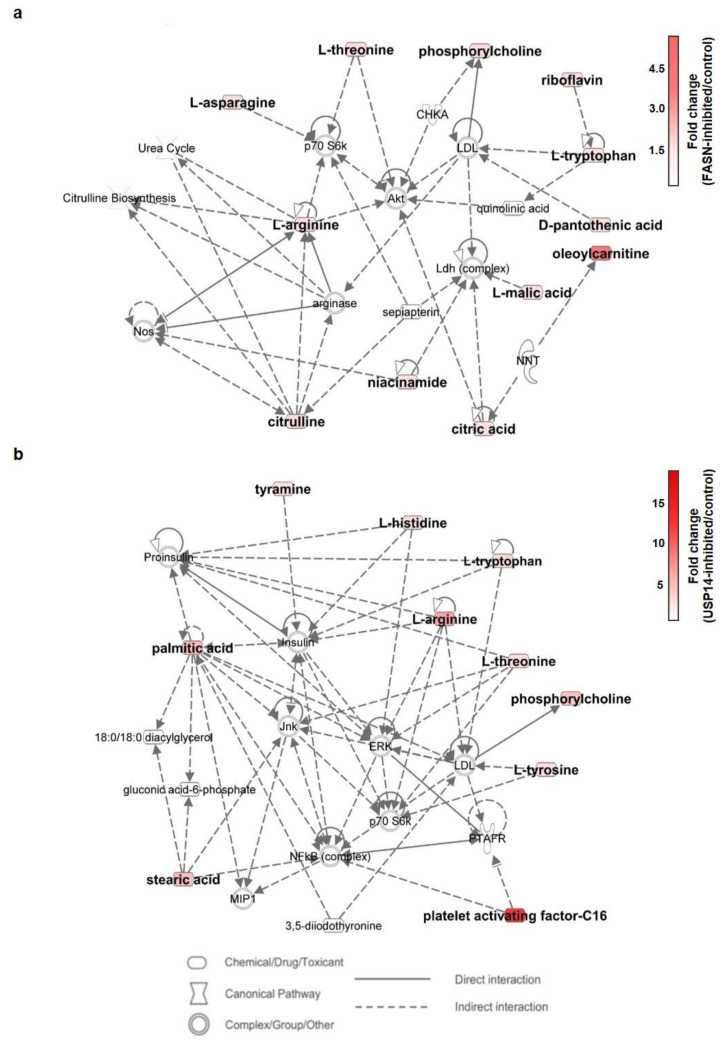
Metabolic network analysis using Ingenuity Pathway Analysis (IPA) software. The network shows altered metabolites involved in (**a**) FASN-inhibited and (**b**) USP14-inhibited LNCaP cells. The red nodes indicate the upregulated molecules, and the intensity indicates the fold change. The specific metabolites involved in network (**a**) are asparagine, pantothenic acid, citric acid, riboflavin, arginine, citrulline, malic acid, niacinamide, and oleoylcarnitine. In network (**b**), they are histidine, palmitic acid, stearic acid, tyramine, and PAF-C16. The two networks show different examples of small molecule biochemistry and molecular transport.

Through identification of the significant metabolite alterations incurred by IU1 and Fasnall, we performed pathway analysis using Ingenuity Pathway Analysis (IPA) software. As shown in Figure 8a,b, the presented pathways involved in each drug show an apparent difference. With the abovementioned PCA scores, this result reiterates the difference in the metabolic impact between the two drugs.

## 3. Discussion

FASN is an important enzyme that involves de novo lipogenesis to produce palmitate, an important building block of more complex lipid species that comprise the cellular lipid pool [1,31]. Since lipid molecules such as phospholipids are important for forming cell membranes, it is not surprising that FASN is overexpressed in various cancer cells, promoting their rapid proliferation. However, although FASN has been a therapeutic target for cancer for a few decades, FASN blockade has yet to be successful in clinical settings. To overcome the limitations of FASN inhibitors as anticancer drugs, we speculated that simultaneously inhibiting the level and the activity of FASN could more potently suppress cancer cell growth. In fact, combination therapy using two or more drugs targeting multiple enzymes has been a foundation for combating cancer [32]. Based on a previous finding that Usp14 is a novel DUB for FASN and enhances FASN stability by blocking proteasomal degradation in MPHs, we expected that a USP14 inhibitor IU1 could further reduce the activity of FASN by interfering with FASN stability when used together with a FASN inhibitor. Surprisingly, our study showed completely unexpected results. Inhibition of USP14 by IU1 or siRNAs targeting USP14 did not reduce FASN levels but rather increased FASN levels and activity in cancer cells. In some cases, DUBs are known to negatively regulate certain protein levels. For example, USP4 targets and deubiquitinates hyaluronan synthase 2 (HAS2), but USP4 does not maintain the stability of HAS, and, rather, loss of USP4 increases hyaluronan synthesis [33]. USP11 inhibits KLF4 expression by cleaving K63-linked polyubiquitin chains in HepG2 hepatocarcinoma cells [34]. In addition, overexpression of USP14 promotes I-κB degradation despite decreasing polyubiquitinated I-κB levels in MLE12 lung cancer cells [35]. From our data, we speculated that USP14 might regulate the protein levels of FASN indirectly through some intermediate proteins. Further studies should be conducted to find direct targets of USP14 that can control FASN levels in cancer cells. However, our study did not fully uncover why the same target is regulated differently by the same DUB in a different environment such as in the hepatocytes versus in the cancer cells. We assumed two possibilities: (1) In cancer cells, the expression of enzymes participating in de novo lipogenesis is excessively increased by metabolic reprogramming, and the proteolytic mechanism by the ubiquitin/proteasome system also fails to function normally. (2) It is not the main function of USP14 to regulate the stability of FASN (it belongs to the minor category among several functions); that is, changes in FASN levels may be an indirect effect of other proteins controlled by USP14, as the function of USP14 in cancer cells is focused on its main target.

If USP14 negatively affects FASN levels in cancer cells, it could be predicted that inhibition of both USP14 and FASN using siRNAs or inhibitors would restore reduced cancer cell viability by inhibiting only FASN. However, USP14 inhibition did not affect the FASN-mediated decrease in cell viability (Figure 1). Additionally, IU1 treatment did not dramatically change the FASN levels. Hence, we hypothesized that the regulation of FASN levels by USP14 is a minor indirect effect and that the regulation of cancer proliferation by USP14 is through a pathway other than the FASN-controlled de novo lipogenesis pathway. Through a metabolomic analysis, we found that inhibition of FASN demonstrated very different metabolic profiles from inhibition of USP14, indicating that these enzymes have little association with cancer cell metabolism. As shown in Figure 6, the PCA plot clustering revealed that two drugs, Fasnall and IU1, have different modes of metabolic variability in cancer cells. To further explore the differences between the two drugs, the heatmap in Figure 7 exhibited the identified metabolites that were significantly altered and are distinguished between the groups. Unlike Fasnall, IU1 did not lower the level of palmitic acid. If IU1 destabilizes FASN and lowers the total protein level, palmitic acid levels should have been lowered. Figure 8 discloses that the two drugs do not share common metabolic networks and show different small molecule biochemistry.

Xu et al. reported that the activation of USP14 by AKT-mediated phosphorylation leads to inhibition of the ubiquitin/proteasome system (UPS), which regulates global protein degradation through the proteasome [36]. Indeed, the ubiquitinome analysis revealed that USP14 can play a role in multiple previously less well-recognized cellular functions, such as energy metabolism, growth factor receptor, and the PI3K-AKT pathway [22]. AKT is an important kinase that regulates various intracellular signaling such as cell proliferation, metabolism, and tumorigenesis. It was demonstrated that AKT-mediated phosphorylation of USP14 may provide a novel mechanism to control multiple signaling processes, including inhibition of the UPS through USP14 activation [36]. Thus, to fully understand the signaling pathways and the interactomes of USP14 involved in cancer cell growth and metabolism, further research on the phosphorylation of USP14 by AKT along with inhibition and knockdown studies of USP14 may be necessary. In future studies, the investigation of alterations in the transduction pathway of USP14 and FASN using transcriptomic analysis is warranted to understand not only the interaction between the two proteins but also the correlation of the interactomes.

Taken together, the results reveal that USP14 negatively regulates FASN levels unexpectedly in the cancer cells, and as a result, inhibition of USP14 was not conducive to cancer cell death through inhibition of FASN. Currently, the development of drugs targeting DUB as a cancer treatment is being actively carried out. However, our results suggest that the role of DUB in cancer may be different from the current understanding, and a more cautious approach is required for its use in combination with other drugs as a target for cancer treatment.

## 4. Materials and Methods

### 4.1. Materials

LC-MS-grade methanol was purchased from Burdick & Jackson (SK Chemicals, Ulsan, Korea). Ultrapure water (18.2 MΩ∙cm) was obtained using a Milli-Q apparatus from Millipore (Burlington, MA, USA). Formic acid and reserpine were all purchased from Sigma-Aldrich.

### 4.2. Cell Culture and Transfection

HEK293T cells, LNCaP cells, MCF7 cells, and A549 cells were purchased from Korea Cell Line Bank (KCLB, Daejeon, Korea). HEK293T cells were cultured in Dulbecco’s modified Eagle’s Medium (DMEM), and LNCaP cells, MCF7 cells, and A549 cells were cultured in Roswell Park Memorial Institute 1640 (RPMI 1640) containing 10% fetal bovine serum, 100 units/mL penicillin, and 100 μg/mL streptomycin (GenDEPOT, Katy, TX, USA). All the cells were maintained at 37 °C in 5% CO_2_. For plasmid transfection, 2 M CaCl_2_ and 2X HBS buffer (50 mM HEPES, 10 mM KCl, 12 mM glucose, 280 mM NaCl, 1.5 mM Na_2_HPO_4_, pH 7.05) were used in HEK293T cells, and Effectene (Qiagen, Hilden, Germany) was used in LNCaP cells following the manufacturer’s instructions. For siRNA transfection, Lipofectamine^TM^ 3000 (Invitrogen, Waltham, MA, USA) was used following the manufacturer’s instructions.

### 4.3. Plasmids and siRNAs

pcDNA3.1-FASN- 6X His was provided by Addgene. Human USP14 was cloned into the 3X Falg-pCMV^TM^_7.1_ vector, and ubiquitin was cloned into the pCS2-HA vector. Control siRNA and siRNA targeting *USP14* (USP14i) or *FASN* (FASNi) were synthesized from Bioneer (Seoul, Korea). siRNA sequences were as follows: *USP14*-**#**1, 5′- AGAAAUGCCUUGUAUAUCAUU-3′, *USP14*-#2, 5′- GGAGAAAUUUGAAGGUGUA-3′, *USP14*-#3, 5′-GCAGCCCUUAGAGAUUUGU-3′; *FASN-*#1, 5′-AACAGCCTCTTCCTGTTCGAC-3′, *FASN*-#2, 5′-CCCUGAGAUCCCAGCGCUG-3′, *FASN* -**#**3, 5′-GGUAUGCGACGGGAAAGUA-3′

### 4.4. Western Blot Analysis

After harvest, cells were lysed using protein lysis buffer (20 mM Tris (pH 7.5), 150 mM NaCl, 1% Triton X-100, 1 mM EDTA, 1 mM EGTA, 2.5 mM sodium pyrophosphate, 50 mM NaF, 5 mM β-glycerophosphate, 1 mM Na_3_VO_4_, protease inhibitor). Western blot analysis was performed using 10–50 μg protein extracts from cells, and protein concentration was measured by using a Micro BCA^TM^ protein assay kit (Thermo Fisher Scientific, Waltham, MA, USA) with a standard curve using BSA. Antibodies that were used for Western blot analysis were as follows: rabbit anti-USP14 (A300-920A, Bethyl Laboratories, Montgomery, TX, USA, 1:2000), rabbit anti-FASN (#3180S, Cell Signaling Technology, Danvers, MA, USA, 1:1000), mouse anti-HA (sc-7392, Santa Cruz Biotechnology, Dallas, TX, USA, 1:1000), mouse anti-Flag (F1804-1MG, Sigma-Aldrich, 1:1000), and rabbit anti-His (#2365S, Cell Signaling Technology, 1:1000). Rabbit anti-β-actin (LF-PA020, AbFrontier, Seoul, Korea, 1:5000) and mouse anti-HSP90α/β (sc12119, Santa Cruz Biotechnology, 1:5000) were used for loading control. An Ez-Capture MG imaging system (ATTO Corporation, Amherst, NY, USA) was used to detect each protein.

### 4.5. Immunoprecipitation

HEK293T cells were transfected with various plasmids as indicated for 24 h. Cells were harvested and lysed by using protein lysis buffer. About 4 mg of lysates was incubated with Flag-agarose beads (Thermo Fisher Scientific, Waltham, MA, USA) overnight at 4 °C. Immuno-complexes were washed with washing buffer (20 mM Tris-Cl, 150 mM NaCl, 1% Triton X-100, 1 mM EDTA, 1 mM EGTA, 2.5 mM sodium pyrophosphate, 50 mM NaF, 5 mM β-glycerophosphate, 1 mM Na_3_VO_4_, pH 7.5) five times and then eluted using 3X Flag peptide (A36805, Thermo Fisher) for 30 min at 4 °C. Before loading to SDS-PAGE, samples were boiled in 6X SDS sample buffer for 5 min. All samples were detected by Western blot analysis using indicated antibodies, and 5% of the samples were used to identify the efficiency of immunoprecipitation.

### 4.6. Ubiquitination Assay

Cells transfected with HA-ubiquitin and tagged plasmids (Flag-USP14 and His-FASN) were treated w/wo 10 μM USP14 inhibitor IU1 (MedChemExpress, Monmouth Junction, NJ, USA) for 24 h. Before harvesting, cells were treated with 10 μM MG132 for 6 h. The cells were lysed with urea lysis buffer (8 M urea, 300 mM NaCl, 50 mM Na_2_HPO_4_, 50 mM Tris, 1 mM PMSF, 10 mM Imidazole, pH 8) and sonicated. About 5 mg of lysates was incubated with HA-agarose beads (Thermo Fisher) or Ni-NTA agarose (Qiagen, Hilden, Germany) for 6 h at 4 °C. The beads were washed with urea washing buffer (8 M urea, 300 mM NaCl, 50 mM Na_2_HPO_4_, 50 mM Tris, 1 mM PMSF, 20 mM Imidazole, pH 8) five times and eluted in 6X SDS sample buffer. Samples were detected by the same procedure indicated for immunoprecipitation.

### 4.7. Cell Viability Assay

LNCaP cells were seeded at a density of 1 × 10^4^ cells/well in a 96-well plate. Cells were transfected with USP14i or FASNi alone or together both for 72 h, or treated with IU1 or Fasnall (Cayman Chemical, Ann Arbor, MI, USA) alone or together both for 48 h. To measure cell viability, a WST-1 assay was performed using EZ-Cytox (DoGenBio, Seoul, Korea) following the manufacturer’s instructions. The absorbance was measured on a microplate spectrophotometer (BioTek, Winooski, VT, USA) at a wavelength of 450 nm.

### 4.8. Enzyme Activity Assay

Into USP14 knockdown and USP14 overexpressed LNCaP cell pellets, 400 μL of phosphate buffer (pH 6.6) was added, and the freeze/thaw cycle was performed three times. The sample was then centrifuged for 10 min at 14,000 rpm to obtain cell lysate. Cell lysate (60 μL), NADPH (20 mM) (5 μL), malonyl-CoA (8 mM) (5 μL), acetyl-CoA (5 mM) (5 μL), and phosphate buffer (25 μL) were pipetted into the 96-well plate. The absorbance was measured for 40 min at 37 °C using a Versa Max 96-well plate reading spectrophotometer (Molecular Devices, San Jose, CA, USA) at 340 nm.

### 4.9. Metabolite Extraction

Ice-cold 80% methanol (100 μL) containing an internal standard (reserpine at a concentration of 2 μg/mL) was used to extract the metabolites. The extract solution was directly added to the cells after PBS washing. Cell pellets were lysed three times by freeze/thaw using liquid nitrogen, and then the lysate was centrifuged for 10 min at 14,000 rpm. Subsequently, 10 μL of supernatant was injected into an Ultimate 3000 UHPLC system LTQ Orbitrap Velos ProTM mass spectrometer (Thermo Fisher Scientific, Waltham, MA, USA) system. A quality control (QC) sample was made by pooling equal volumes of each sample. Before the analysis, conditioning for the machine was performed by injecting QC samples 10 times before runs. We also analyzed the QC samples in addition to 10 analytical sample runs to assess the repeatability of the instrument.

### 4.10. Metabolomic Analysis

Metabolic profiling was conducted using an Ultimate 3000 UHPLC system, coupled to an LTQ Orbitrap Velos Pro mass spectrometer (Thermo Fisher Scientific, Waltham, MA, USA). An ACQUITY UPLC HSS T3 column (2.1 × 100 mm, 1.8 μm; Waters) was used at 40 °C and flow rates of 0.4 mL/min. Gradient elution using mobile phase A (0.1% formic acid in distilled water) and mobile phase B (0.1% formic acid in methanol) was performed [37]. All samples were randomly analyzed to avoid the potential effects of the analysis sequence. MS using an electrospray ionization (ESI) source functioned in positive and negative ionization modes. Gradient elution was carried out at a flow rate of 0.4 mL/min using mobile phase A (0.1% formic acid in distilled water) and B (0.1% formic acid in methanol). After maintaining initial conditions for 2 min, a linear gradient that reached 100% B over 14 min was applied and held for 1 min at 100% B. All samples were analyzed randomly to eliminate the effects of analysis order. The capillary voltages of positive and negative modes were +3.2 kV and −2.5 kV, respectively, and the cone voltage was 40 V for both polarities. The mass range of 50–1200 Da was obtained by data-independent centroid mode.

### 4.11. Data Preprocessing and Statistical Analysis for Identification of Metabolites

The data were initially processed using Compound Discoverer 2.1 software (Thermo Scientific) for peak deconvolution and data normalization. For statistical analysis, data were normalized to the internal standard and DNA concentrations of each sample. The concentration of DNA in each sample was quantified using a Nano-MD (SINCO). Metabolic differences between groups were assessed by a multivariate statistical analysis using the partial least squares discriminant analysis (PLS-DA) algorithm using EZinfo software of the Masslynx V4.1 workstation (Waters, Milford, MA, USA. The quality of the PLS-DA model was evaluated by cross-validation parameters, R2 and Q2 [38]. Molecular identification and structure prediction were performed based on the exact mass retention time pairs using Xcalibur™ software (Thermo Finnigan). The acquired chromatograms after analyzing the cell pellet were subjected to Compound Discoverer™ software (Thermo Scientific) to obtain the intensity and *m/z* value of each peak.

The identities of peaks were verified by an in-house database and online databases, specifically Human Metabolome Database (http://www.hmdb.ca/ accessed on 15 May 2021), METLIN (http://metlin.scripps.edu/ accessed on 15 May 2021), Chemspider (http://www.chemspider.com/ accessed on 22 May 2021), and KEGG (http://www.genome.jp/kegg/ accessed on 22 May 2021). The heatmap was statistically evaluated by Wilcoxon–Mann–Whitney tests, which showed differences in cellular levels of each metabolite using MetaboAnalyst 4.0 (http://www.metaboanalyst.ca/ accessed on 12 June 2021). Moreover, the Student’s *t*-test was used to explore the altered metabolite features between the samples. *; *p* < 0.05, **; *p* < 0.01, ***; *p* < 0.001, ****; *p* < 0.0001.

### 4.12. Ingenuity Pathway Analysis

Ingenuity Pathway Analysis (IPA) (http://www.ingenuity.com/ accessed on 20 July 2021) was performed to explore the changed metabolic pathways. IPA could identify the metabolic pathways that significantly changed after drug treatment. The metabolic pathway networks were built, and the results showed the alteration associated with the mechanisms relevant to Fasnall and IU1.

### 4.13. Statistical Analysis

Results are shown as means ± standard deviations of at least three independent experiments unless otherwise indicated in the figure legends. All statistical analyses were obtained using Student’s *t*-tests. Values of *p* < 0.05 were considered statistically significant.

## Supplementary Materials

The following are available online at https://www.mdpi.com/article/10.3390/ijms222413437/s1.

## Appendix A

**Table A1:** Significantly altered metabolites after the drug treatment. Student’s *t*-test was performed to evaluate the statistical significance of changes in metabolite levels. Bold indicates that the changed metabolites are statistically significant.

Metabolite	Fasnall			IU1			Fasnall + IU1		
	*p* -Value	Fold Change	Trend	*p* -Value	Fold Change	Trend	*p* -Value	Fold Change	Trend
Asparagine	<0.001	**1.55**	up	NS	1.18	up	<0.0005	**2.892**	up
Citric acid	<0.001	**0.92**	down	NS	1.36	up	<0.005	**0.55**	down
Citrulline	<0.05	**1.04**	up	NS	1.11	up	<0.0005	**0.61**	down
Glutamic acid	NS	0.94	down	NS	0.94	down	<0.005	**0.616**	down
Glutathione	NS	**0.98**	down	NS	0.93	down	<0.0001	**0.493**	down
Arginine	<0.01	**1.52**	up	<0.01	**4.7**	up	<0.05	**4.014**	up
Leucine	NS	2.82	up	NS	1.42	up	<0.05	**2.318**	up
Histidine	<0.0001	**1.15**	up	<0.05	**1.4**	up	<0.01	**1.997**	up
Threonine	<0.0001	**2.24**	1.24	<0.05	**1.26**	up	<0.01	**1.686**	up
Tryptophan	<0.01	**2.51**	up	<0.01	**1.96**	up	<0.0001	**5.028**	up
Tyrosine	<0.05	**1.42**	up	NS	1.26	up	<0.001	**2.36**	up
N-Acetylaspartylglutamic acid	<0.001	**1.14**	up	NS	0.98	down	NS	0.959	down
N-Acetyl-DL-alanine	<0.0001	**1.23**	up	NS	1.03	up	<0.0001	**1.651**	up
N-Acetyl-DL-aspartic acid	NS	1.04	up	<0.05	**0.9**	down	<0.05	**0.858**	down
N-Acetyl-DL-glutamic acid	NS	0.98	down	NS	1.05	up	NS	0.474	down
Nicotinamide	<0.001	**0.94**	down	<0.05	**0.71**	down	<0.001	**0.475**	down
N-Methylglutamic acid	<0.05	**2.28**	up	<0.05	**1.78**	up	<0.0001	**4.728**	up
Pantothenic acid	<0.05	**1.12**	up	NS	0.87	down	<0.05	**1.148**	up
Tyramine	<0.05	**1.81**	up	<0.05	**1.47**	up	<0.05	**2.048**	up
Saccharopine	<0.001	**1.15**	up	<0.05	**1.29**	up	NS	1.058	up
2-Furoglycine	<0.05	**1.04**	up	NS	1.02	up	<0.05	**0.801**	down
12-Hydroxydodecanoic acid	<0.001	**15.32**	up	NS	0.74	down	<0.0001	**53.337**	up
9-oxo-10E,12Z-octadecadienoic acid	NS	1.42	up	NS	0.35	down	NS	5.502	up
Arachidonic acid	<0.001	**1.29**	up	NS	0.44	down	<0.05	**7.322**	up
Adrenic acid	NS	0.96	down	<0.0001	**0.56**	down	<0.05	**1.996**	up
Arachidonic acid-(2-aminoethyl)-ester	NS	1.41	up	<0.05	**2.08**	up	NS	1.718	up
Dihomo-alpha-linolenic acid	<0.001	**1.16**	up	<0.005	**0.43**	down	<0.05	**3.825**	up
Dihomo-gamma-linolenic acid	NS	2.65	up	NS	5.12	up	NS	3.153	up
Palmitic acid	NS	1.32	up	<0.0001	**3.63**	up	<0.001	**2.044**	up
Stearic acid	<0.001	**1.37**	up	<0.0001	**3.43**	up	<0.05	**2.046**	up
Oleoyl-L-carnitine	<0.0001	**31.96**	6.922	NS	1.56	up	<0.0001	**27.781**	up
Palmitoyl-L-carnitine	<0.001	**9.85**	up	<0.05	**2.89**	up	<0.0001	**39.377**	up
Riboflavin	<0.05	**1.23**	up	NS	1.2	up	<0.05	**1.335**	up
6-Hydroxyflavone	<0.001	**1.36**	up	NS	1.57	up	<0.05	**2.479**	up
Glycerophosphocholine	<0.01	**0.83**	down	NS	1.12	up	<0.001	**0.286**	down
2-deoxy-2-thio Arachidonoyl PC	<0.05	**1.01**	up	NS	1.2	up	NS	1.268	up
LysoPC(18:1/0:0)	<0.001	**1.14**	up	<0.0001	**3.74**	up	<0.001	**1.821**	up
LysoPC(18:0/0:0)	<0.001	**1.65**	up	<0.0001	**7.87**	up	<0.05	**3.069**	up
LysoPE(16:0/0:0)	<0.0001	**1.46**	up	<0.0001	**2.46**	up	<0.001	**3.355**	up
LysoPE (22:6/0:0)	NS	1.12	up	NS	1.04	up	<0.05	**1.325**	up
LysoPE alkenyl (18:1)	<0.001	**1.19**	up	<0.05	**1.34**	up	<0.05	**1.431**	up
PC (18:1/2:0)	<0.001	**1.2**	up	<0.0001	**10.76**	up	<0.001	**2.914**	up
PC (30:1)	<0.005	**1.21**	up	NS	1.13	up	<0.05	**1.357**	up
PC (36:5)	NS	0.673	down	NS	0.56	down	NS	0.509	down
PC (16:0/0:0)	<0.0001	**1.16**	up	<0.0001	**5.15**	up	<0.0001	**2.119**	up
PE (18:1/0:0)	<0.0001	**2.28**	up	<0.0001	**2.95**	up	<0.05	**1.649**	up
Malic acid	<0.05	**1.04**	up	NS	0.87	down	NS	0.892	down
5’-Deoxy-5’-methylthioadenosine	<0.05	**0.97**	down	<0.05	**1.33**	up	NS	1.178	up
Adenosine monophosphate (AMP)	<0.001	**1.49**	up	<0.05	**1.48**	up	<0.001	**3.897**	up
Guanosine monophosphate (GMP)	<0.0001	**1.49**	up	<0.05	**0.64**	down	<0.001	**2.445**	up
Uridine monophosphate (UMP)	<0.05	**1.42**	up	NS	1.1	up	<0.05	**2.365**	up
Taurine	<0.001	**2.13**	up	NS	1.17	up	<0.0001	**3.387**	up
Phosphocholine	<0.0001	**2.134**	up	<0.01	**3.1**	up	<0.05	**4.275**	up
Hydroxyphenyllactic acid	<0.01	**2.7**	up	<0.05	**1.51**	up	<0.0001	**5.856**	up
Cholic acid	<0.05	**1.37**	up	NS	1.21	up	<0.0001	**1.944**	up
