# Supplementary material for: USP14 Regulates Cancer Cell Growth in a Fatty Acid Synthase-Independent Manner
Source: Int J Mol Sci. 2021 Dec 14;22(24):13437. doi: 10.3390/ijms222413437 (PMC8707130; doi:10.3390/ijms222413437)
Supplement: Supplementary file 1 [file ijms-22-13437-s001.zip › ijms-1477075-supplementary.pdf]

USP14 regulates cancer cell growth in a fatty acid synthase-independent manner.

Ji Su Yang [1,2], Naeun Yoon [1,3], Mingyu Kong [1,4], Byung Hwa Jung [1,5], Hyunbeom Lee [1,6,*] and Jinyoung Park [1,*]

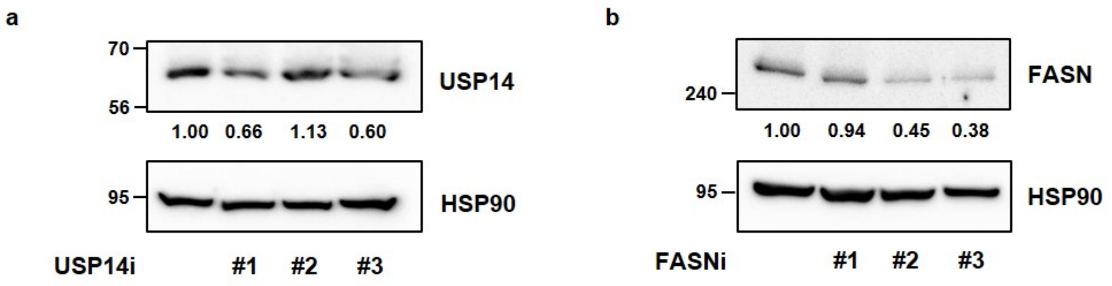

Figure S1. Efficiency of siRNAs targeting USP14 or FASN. LNCaP cells were transfected with (a) siRNAs targeting *USP14* (USP14i-#1, #2, #3) or (b) *FASN* (FASNi-#1, #2, #3) for 48 h. Cell lysates were immunoblotted with the indicated antibodies. HSP90 was used as a loading control. The band intensity of USP14 or FASN was measured by Image J and then normalized by each HSP90 protein.

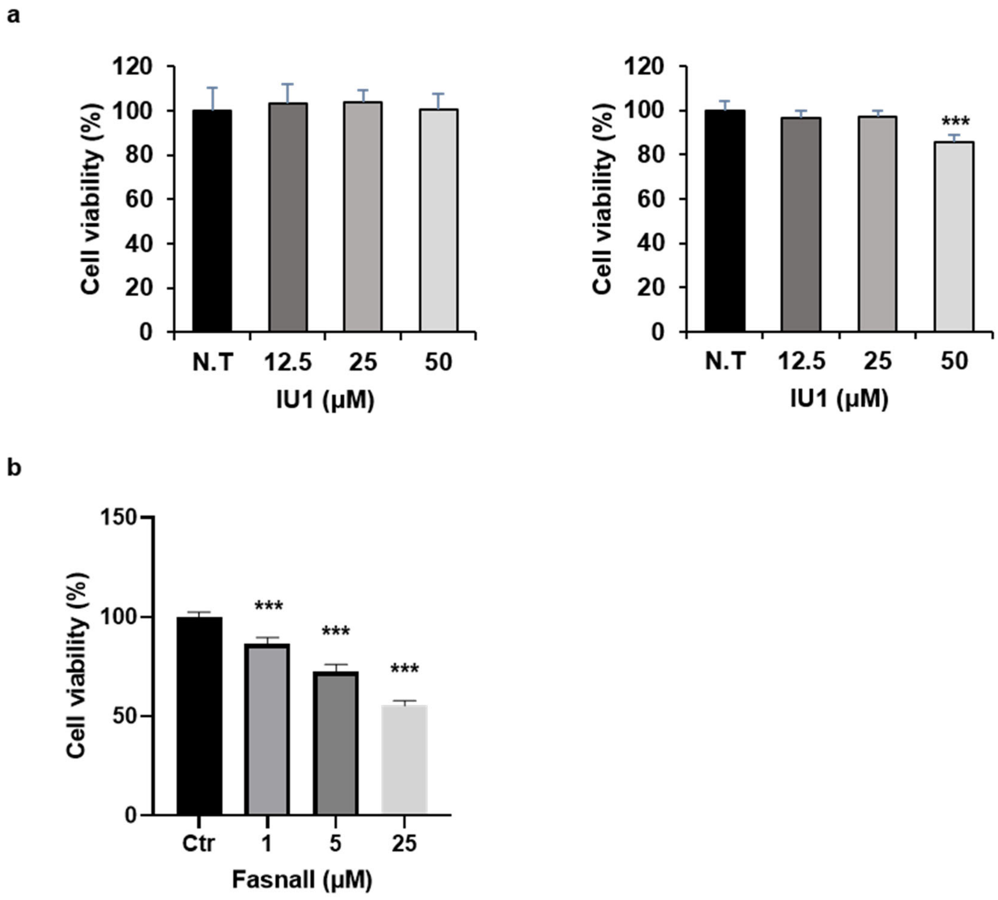

Figure S2. IU1 or Fasnall reduces cancer cell proliferation. (a, b) Cell viability was measured by the WST-1 assay. LNCaP cells were treated with (a) IU1 for 24 h (left) or 48 h (right), or (b) Fasnall for 24 h.

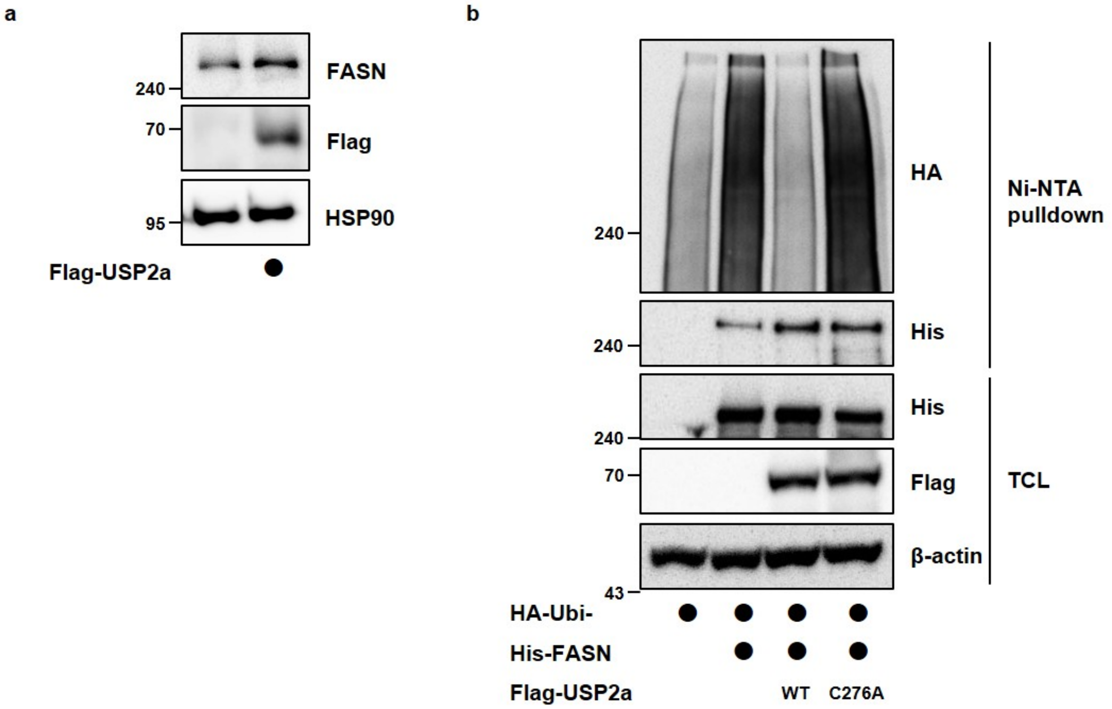

Figure S3. USP2a increases FASN protein levels by inhibiting FASN ubiquitination in LNCaP cells. (a) LNCaP cells were transfected with Flag-USP2a. (b) LNCaP cells were transfected with HA-ubiquitin alone or in combination with His-FASN, Flag-USP2a WT or Flag-USP2a C276A. 10 μM MG132 treated for 6 h in cells before the harvest. FASN ubiquitination was observed using a Ni-NTA-mediated pull down assay. Cell lysates were immunoblotted with the indicated antibodies.
